# Crystal Structure of *Bombyx mori* Lipoprotein 6: Comparative Structural Analysis of the 30-kDa Lipoprotein Family

**DOI:** 10.1371/journal.pone.0108761

**Published:** 2014-11-07

**Authors:** Agnieszka J. Pietrzyk, Anna Bujacz, Malgorzata Łochynska, Mariusz Jaskolski, Grzegorz Bujacz

**Affiliations:** 1 Center for Biocrystallographic Research, Institute of Bioorganic Chemistry, Polish Academy of Sciences, Poznan, Poland; 2 Institute of Technical Biochemistry, Faculty of Biotechnology and Food Sciences, Technical University of Lodz, Lodz, Poland; 3 Institute of Natural Fibers and Medicinal Plants, Poznan, Poland; 4 Department of Crystallography, Faculty of Chemistry, A. Mickiewicz University, Poznan, Poland; NCI-Frederick, United States of America

## Abstract

The 30-kDa lipoprotein (LP) family of mulberry silkworm comprises major hemolymph proteins specific to the fifth instar larvae. The family consists of 46 members, 24 of which are referred to as typical 30-kDa LPs. To date, two crystal structures of 30-kDa LPs from *Bombyx mori* have been described (Bmlp3 and Bmlp7). Here, we present the crystal structure of Bmlp6, another 30-kDa LP member. Bmlp6 is comprised of two domains characteristic of this family, the VHS-type N-terminal domain and β-trefoil C-terminal domain. The structures of the three 30-kDa LPs have been compared and a number of differences are noted, including loop conformation, the surface electrostatic potential, and the potential binding cavities. We discuss the observed structural differences in the light of the potential different roles of the particular 30-kDa LP members in silkworm physiology.

## Introduction

The mulberry silkworm, *Bombyx mori*, is an insect of high economical importance due to its ability to produce natural silk fibers. It was the first insect within the order *Lepidoptera* for which draft genomic sequence was completed in 2004, independently by a Chinese [1] and Japanese [2] groups. In 2008, The International Silkworm Genome Consortium was formed by these groups and all existing data were merged and supplemented with newly obtained sequences [3]. The genome information is accessible via the knowledgebase for silkworm biology and genomics, SilkDB [4], [5]. Despite these efforts, the information about silkworm genes and proteins is still not well ordered in international databases. For example, there are significant differences in the annotations and even in the sequences of particular proteins. This is especially true of 30-kDa lipoproteins (30-kDa LPs), the most abundant group of proteins found in the hemolymph of the silkworm fifth instar larvae [6]–[9]. The first cDNA clones of 30-kDa LPs with distinct mRNA were isolated by Sakai et al. [8] and annotated as PBMHP-6, PBMHP-12, PBMHPC-19, PBMHPC-21 and PBMHPC 23 or LP(1–5), respectively [8]. Later, according to the draft of silkworm genomic sequence [1], [2], ten genes of 30-kDa LPs were predicted and denoted as Bmlp1-10 [10]. A few years later, a more detailed analysis based on the completed silkworm genomic sequence revealed the presence of 46 genes of 30-kDa LPs in the silkworm genome [11]. They were annotated as Bmlp1–46 according to the previous terminology rules [10]. The 30-kDa LPs can be divided into three subfamilies: typical 30-kDa LPs (Bmlp1–24), serine/threonine-rich LPs (Bmlp25–36) and ENF peptide-binding proteins (Bmlp37–46) [11].

An analysis of expressed sequence tags (ESTs) indicated that the level of 30-kDa LPs expression is variable for the different genes. The highest expression level was observed for *Bmlp7, Bmlp3, Bmlp2, Bmlp1* and *Bmlp9*, listed in the decreasing order of the number of ESTs [10]. Moreover, proteomic studies of silkworm hemolymph [12]–[15] and of the fat bodies [14], the site of 30-kDa LP synthesis, revealed that the expression of particular genes varies from the end of the fourth to the end of the fifth instar. Although 30-kDa LPs are usually described as proteins specific to the fifth instar larvae [7], some members of this family, e.g. Bmlp20, are expressed only at the end of the fourth instar [15].

The 30-kDa LPs are involved in many physiological processes in the silkworm body. They transport lipids in the hemolymph [16], tryptophan metabolites and pigments in diapause silkworm eggs [17], are involved in an immune response pathway in an antifungal defense system [18], [19], serve as the source of nutrients during pupation and adult development [13], [20], and are the second major yolk protein component [21]–[23].

The 30-kDa LPs can be also very useful laboratory tools due to their anti-apoptotic properties. An addition of silkworm hemolymph to different cell cultures inhibits apoptosis and improves the viability of the cells. The anti-apoptotic properties of the hemolymph were demonstrated for a number of cell cultures, including insect Sf9 [24]–[27], mammalian CHO [28]–[29], and human HeLa [30] and HEK293 [29] cell lines. 30-kDa LP members were identified as the hemolymph component responsible for those properties [29], [31]. Finally, it was shown that a 30-kDa LP protein is able to penetrate various cell types and can also deliver a cargo protein [32].

Here we present the crystal structure of *Bombyx mori* lipoprotein 6 (Bmlp6). To-date, only two 30-kDa LPs were characterized structurally, Bmlp7 [33], [34] and Bmlp3 [35]. In this paper we also present a detailed comparative structural analysis of the three 30-kDa LPs, and discuss how the structural differences could be related to diverse physiological roles of the members of this large protein family.

## Materials and Methods

### Hemolymph collection, protein purification and crystallization

*B. mori* hemolymph was collected from fifth instar larvae as described previously [36]. Bmlp6 was isolated from the hemolymph using a three-step purification protocol. The first two steps have been already described [36]. Briefly, initial protein separation was carried out using size exclusion chromatography with a Superdex 200 prep grade column (XK 16/100, Amersham Biosciences). The collected fractions containing the 30-kDa proteins were concentrated and applied on a Q Sepharose column (XK 16/10, Amersham Biosciences). Stepwise-elution ion exchange chromatography allowed further separation. The fraction containing Bmlp6 was eluted with 30 mM NaCl. The third purification step was chromatofocusing performed on a MONO P 5/200 GL (GE Healthcare Life Sciences) column equilibrated with 0.025 M Bis-Tris/CH_3_COOH pH 7.0. Prior to sample application, the column was washed with ∼10 ml of diluted mixture of Polybuffer 96 and Polybuffer 74 (in 19 1 ratio) pH 6.0. The same mixture was used as elution buffer. The fractions containing Bmlp6 were concentrated to ∼3 mg/ml.

Crystals of Bmlp6 were grown at 20°C using the hanging drop vapor diffusion method and 10% isopropanol, 10% PEG 3350 and 0.1 M MES pH 6.0 as the precipitating buffer. The crystals were tiny plates and grew in clusters. Initial crystals were grown using the sample obtained after two-step purification. However, the third purification step was crucial to obtain the crystals of good quality and good diffraction properties.

### Data collection and processing

X-ray diffraction data for initial crystals were collected at beamlines BL14.1 and BL14.2 of the BESSY synchrotron, Berlin, Germany [37]. The crystals diffracted only to 7.0 Å resolution. X-Ray diffraction data for Bmlp6 crystals, obtained after the third purification step, were collected at the EMBL beamline P14 of the PETRA III synchrotron at DESY, Hamburg, Germany, using a PILATUS 6M detector. The diffraction images were recorded at 100 K for one single crystal using the oscillation method with a rotation of 0.2° per image. No cryprotection was necessary due to the presence of isopropanol in the crystallization buffer. The crystal was mounted in a nylon fiber loop and vitrified in a stream of cold nitrogen gas. The diffraction images were indexed, integrated and scaled using XDS [38], [39]. The data collection and processing statistics are presented in Table 1.

**Table 1:** Diffraction data collection and refinement statistics.

Crystal data	Bmlp6
Space group	*P*2_1_
Unit cell parameters a, b, c [Å]; α, β, γ [°]	83.2, 85.7, 104.5; 90.0, 104.8, 90.0
Protein molecules/ASU	5
VM [Å^3^/Da]	2.62
Solvent content [%]	53.1
**X-ray data collection**	
Temperature [K]	100
Radiation source	PETRA III, P14 (MX2)
Wavelength [Å]	0.976
Detector	DECTRIS PILATUS 6M
Crystal-detector distance [mm]	388
Rotation range [°]	0.2
Total rotation [°]	360
Exposure/image [s]	0.2
Resolution [Å]	47–1.8 (1.9–1.8)^a^
Mosaicity [°]	0.30
Intensities measured	803 831
Unique reflections	124 752
*R_merge_* b [%]	4.8 (55.1)
Redundancy	6.4 (5.2)
<I/σI>	22.3 (3.5)
Completeness [%]	94.9 (73.0)
**Refinement**	
Resolution [Å]	47–1.8
*R_work_/R_free_* c [%]	16.1/18.3
*R_work_* set count	123 486
*R_free_* test set count	1265
Protein/solvent atoms	9885/1131
R.m.s. deviations (bond lengths) [Å]	0.013
R.m.s. deviations (bond angles) [°]	1.53
<*B*> for protein/solvent [Å^2^]	25.8/43.6
Favored/disallowed Ramachandran φ/ψ angles [%]	92.8/0.0
**PDB code**	4PC4

a Values in parentheses are for the highest resolution shell.

b *R_merge_* =  ∑_h_∑_j_ | I_hj_ - <I_h_> |/∑_h_∑_j_ I_hj_, where I_hj_ is the intensity of observation j of reflection h.

c *R_work_* =  ∑_h_ | | F_o_| - | F_c_| |/∑_h_ | F_o_| for all reflections, where F_o_ and F_c_ are observed and calculated structure factors, respectively. *R_free_* is calculated analogously for the test reflections, randomly selected and excluded from the refinement.

### Structure determination and refinement

The crystal structure of Bmlp6 was determined by molecular replacement (MR) using Phaser-MR [40] and the coordinates of chain A of Bmlp7 (PDB: 4EFP) [34] as a starting model. The final model was completed after several cycles of manual rebuilding in COOT [41] and refinement in REFMAC [42] with the inclusion of TLS parameters [43]. The progress of the refinement was monitored and validated using 1265 reflections set aside for R_free_ testing [44]. The geometry of the model was assessed in PROCHECK [45]. The refinement statistics are summarized in Table 1.

### N-terminal sequencing

Preparation of a Bmlp6 sample for Edman degradation included tricine-SDS gel electrophoretic separation [46] followed by protein transfer to a PVDF Immobilon membrane, PSQ 0.22 µm (Millipore). The single protein band corresponding to the molecular weight of 28 kDa was cut and subjected to Edman degradation cycles, performed using a fully automated Procise 491 (Applied Biosystems) sequencer.

## Results and Discussion

### Protein identification and database-related ambiguities

Bmlp6 was isolated from mulberry silkworm hemolymph as an unknown protein and successfully purified by FPLC using a three-step purification protocol. To facilitate identification, ten N-terminal amino acid residues were sequenced by Edman degradation. The analysis returned the following N-terminal sequence: _1_GVVELSADTS_10_. The final identification of the protein was complicated because of the high level of amino acid sequence similarity among the members of the 30-kDa LP family. The problem was aggravated by the confusing information about silkworm proteins in the international sequence databases. Ultimately, the X-ray crystallographic electron density maps were instrumental for the final identification of the investigated protein. This study is part of a wider project, aimed at structural analysis of the most abundant proteins in the hemolymph of silkworm fifth instar larvae. The investigated proteins are typically isolated as unknown proteins, requiring definitive identification. Previously, we have described our results for Bmlp7 [34], Bmlp3 [35] and for a complex of two arylphorins, SP2-SP3 [47]. In all of those cases, the successful amino acid sequence identification was largely based on electron density maps. An overview of the benefits of sequencing from electron density maps has been presented by Pietrzyk et al. [48].

**Figure 1:**
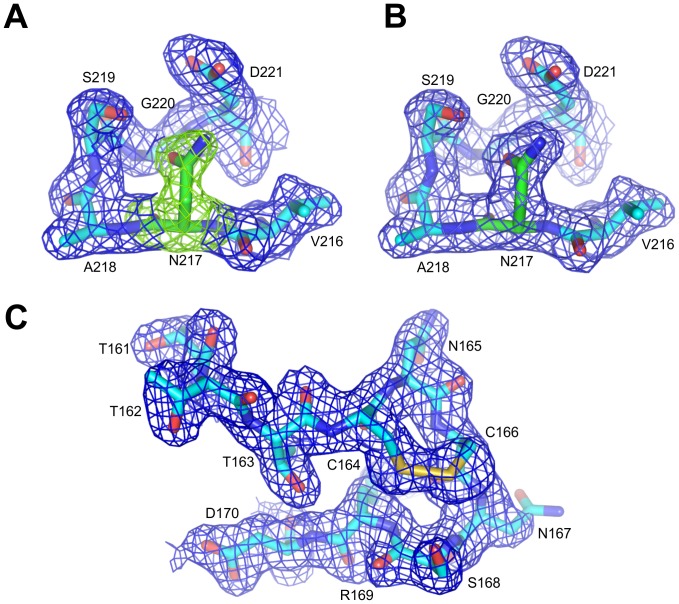
Electron density maps of Bmlp6. (A, B) *Fo-Fc* and *2Fo-Fc* electron density maps (contour 5.0 σ and 1.0 σ, respectively), unequivocally demonstrate that residue 217 of Bmlp6 (green) is not Tyr but Asn. (C) *2Fo-Fc* electron density map (contour 1.0 σ) of a loop (residues 161–170) containing disulfide bridge is presented to illustrate that electron density maps were of a very good quality also at the loop regions. For both fragments the molecule A was arbitrary chosen.

**Figure 2:**
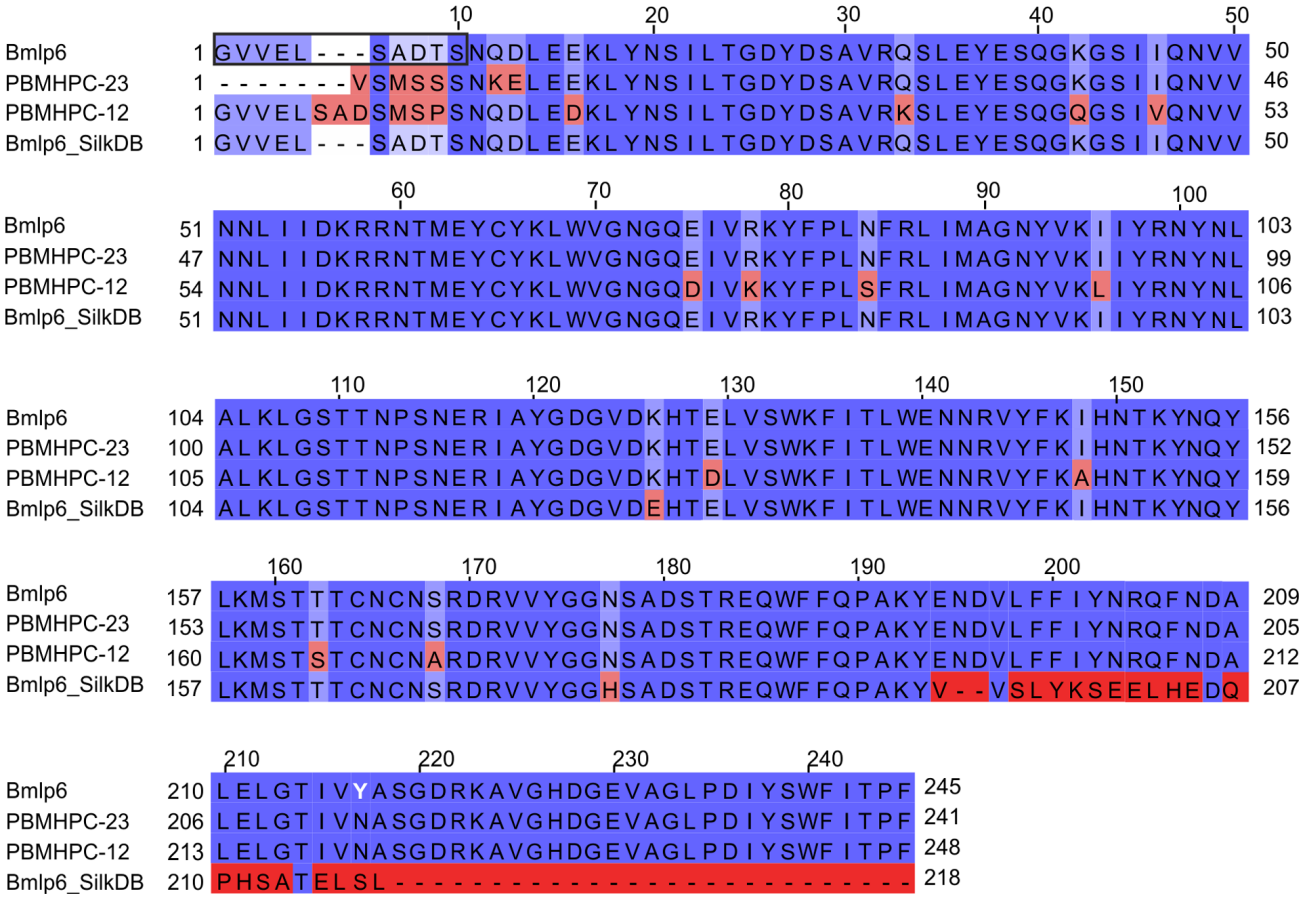
Alignment of sequences corresponding to Bmlp6. Amino acid sequence alignment of Bmlp6 (UniProt: A7LIK7; NCBI-Protein: NP_001095198), PBMHPC-23 (UniProt: P09338), PBMHPC-12 (UniPtot: P09335) and Bmlp6_SilkDB (SilkDB: BGIBMGA004457) calculated in ClustalW (http://www.ebi.ac.uk/Tools/msa/clustalw2/). The alignment is colored according to identity (dark blue) and similarity (light blue) using Jalview (http://www.jalview.org/) [60]. The evident sequencing error at the C-terminus of Bmlp6_SilkDB (highlighted in dark red, starting from position 194) has been disregarded in sequence similarity calculations. The only discrepancy between Bmlp6 (UniProt: A7LIK7) and the amino acid sequence determined by X-ray crystallography is indicated by white font, at position 217. The N-terminal sequence of Bmlp6 established by Edman degradation is boxed. The displayed sequences correspond to mature proteins without signal peptides.

The crystal structure of Bmlp6 was solved at 1.8 Å resolution by molecular replacement using Bmlp7 as a model (PDB ID: 4EFP) [34]. The initial electron density maps were of a good quality. A plausible amino acid sequence of the investigated protein was found, according to the experimental information from the N-terminal sequencing and electron density maps, in two databases, namely in UniProt under the accession code A7LIK7 and annotated as 30K lipoprotein; and in NCBI-Protein under the accession code NP_001095198 and annotated as low-molecular 30-kDa lipoprotein PBMHPC-23 precursor. The only discrepancy between the two sequences and the amino acid sequence deduced from electron density maps is at position 217, which is Asn in the crystal structure (Fig. 1), while according to the sequences in the databases it should be Tyr. A distinction between Asn and Asp at this position was based on the analysis of H-bonding network, the analysis of temperature factors and the fact that Asn residue is present at this position in a number of other 30-kDa LPs. Small sequence variations (e.g. Tyr/Asn) are not unusual when the same protein is isolated from different silkworm strains [14] and may represent genuine genetic polymorphism. In this particular case, a single nucleotide codon mutation can cause the Tyr/Asn substitution. Interestingly, the amino acid sequence of PBMHPC-23 available in UniProt (Accession code: P09338) is incomplete at the N-terminus (Fig. 2). The search for sequences corresponding to PBMHPC-23 was also performed against the SilkDB database [4], [5]. A major part of PBMHPC-23 (starting at the N-terminal residue) corresponds to residues 1-193 of the Bmlp6 sequence found in the SilkDB, but the C-terminal part of the Bmlp6 sequence is incorrect and incomplete, starting at residues 194 and 218, respectively (Fig. 2). However, to be consistent with the current terminology rules introduced by Sun et al. [10], the name of the investigated protein has been assigned as Bmlp6 (despite of the errors in SilkDB) instead of PBMHPC-23, which refers to the nomenclature introduced in 1980s [8]. The highest sequence similarity to Bmlp6 is observed for Bmlp2 (UniProt PBMHP-12, accession code: P09335). The Bmlp6/Bmlp2 amino acid sequences alignment (Fig. 2) indicates 97% similar and 93% identical residues.

Our results indicate that Bmlp6 is the third most abundant 30-kDa LP protein in the hemolymph of the fifth instar larvae. The first and the second most abundant proteins are Bmlp7 and Bmlp3, respectively. The first two steps of purification protocol (size exclusion and ion exchange chromatography) were common for three mentioned 30-kDa LPs and they always provided three samples (corresponding to three different peaks on chromatograms), each containing one of the three 30-kDa LPs as the main component. According to all chromatograms obtained using a number of hemolymph samples, the quantities of the proteins were always the highest for Bmlp7, then for Bmlp3 and Bmlp6. The first (Bmlp7) and the second position (Bmlp3) is in agreement with the EST analysis [10]. However, it was also reported that Bmlp2 is at the third most abundant 30-kDa LP with 413 ESTs and the number of ESTs for Bmlp6 was estimated at only 15 [10]. However, this discrepancy with our data is most likely due to the errors in the sequence databases mentioned above. It is of note that EST analysis is based on short sequence fragments only, whereas our results reflect the intact proteins isolated from their natural source.

### Crystal structure, overall fold and crystal packing of Bmlp6

Bmlp6 crystallized in space group *P*2_1_. The molecular replacement procedure found five copies of the polypeptide chain in the asymmetric unit. The final model was refined to *R/R_free_* factors of 16.1/18.3% for diffraction data between 47.0 and 1.8 Å. According to PROCHECK [45], 92.8% of all residues were in the most favored regions of the Ramachandran plot and no residues had disallowed conformation.

**Figure 3:**
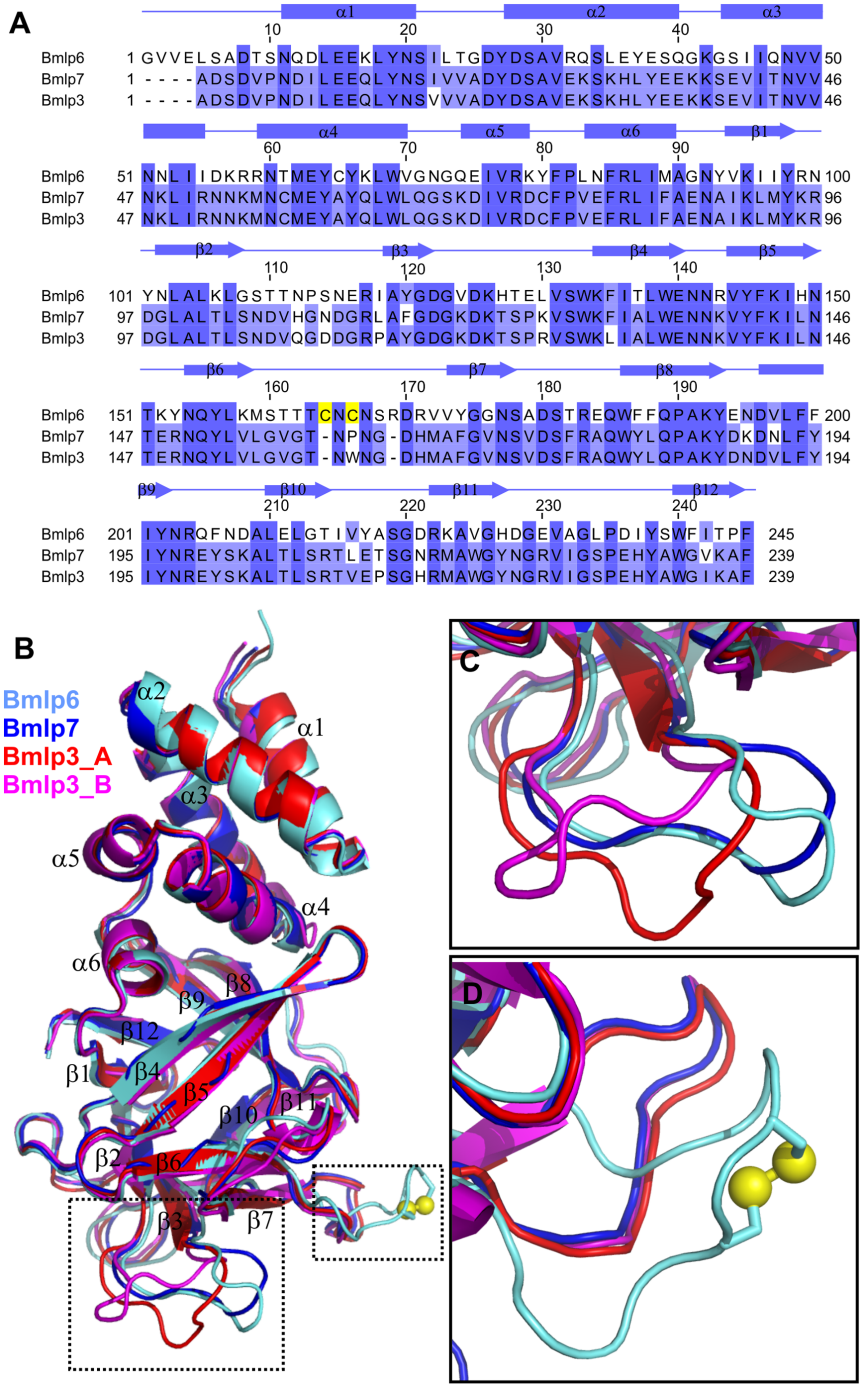
Structural comparison of Bmlp6, Bmlp3 and Bmlp7. (A) Amino acid sequence alignment of Bmlp6 (UniProt: A7LIK7), Bmlp7 (UniProt: E5EVW2) and Bmlp3 (UniPtot: H9J4F6) calculated in ClustalW (http://www.ebi.ac.uk/Tools/msa/clustalw2/). The alignment is coloured according to identity (dark blue) and similarity (light blue) using Jalview (http://www.jalview.org/) [60]. The cysteine residues forming a disulfide bridge are highlighted in yellow. The secondary structure elements are assigned as α-helices (cylinders) and β-strands (arrows). (B) Cα atom superpositions of the crystallographic models of Bmlp6 (chain A, this work), Bmlp7 (PDB: 4EFP, chain A) and Bmlp3 (PDB: 4IY9, chain A and B). (C) The most flexible loop of the CTD among the three compared proteins, located between strands β2 and β3. (D) The Bmlp6 loop containing a disulfide bridge (yellow spheres), located between strands β6 and β7, is compared with the corresponding loops in Bmlp7 and Bmlp3. The color code in panels B–D is light blue for Bmlp6, dark blue for Bmlp7, and shades of red for chains A and B of Bmlp3.

Bmlp6, as a 30-kDa LP member, has a fold (Fig. 3B) characteristic of this protein family, classified as Lipoprotein_11 family (Pfam: PF03260). Thus, Bmlp6 is composed of two domains, the N-terminal VHS domain (Pfam: PF00790) and the C-terminal β-trefoil domain (Pfam: PF14200). The N-terminal domain (NTD) of Bmlp6 (residues 1-91) consists of six helices forming a right-handed superhelix and packed in a globular form. The C-terminal domain (CTD) of Bmlp6 (residues 92–245) is folded as a ricin-type β-trefoil lectin-like domain. The β-strands forming the trefoil are connected by an intricate system of loops, all of which are well-defined in electron density. A detailed comparison of the NTD and CTD domains of the 30-kDa LPs with other structures available in the PDB was presented previously [33], [34].

**Figure 4:**
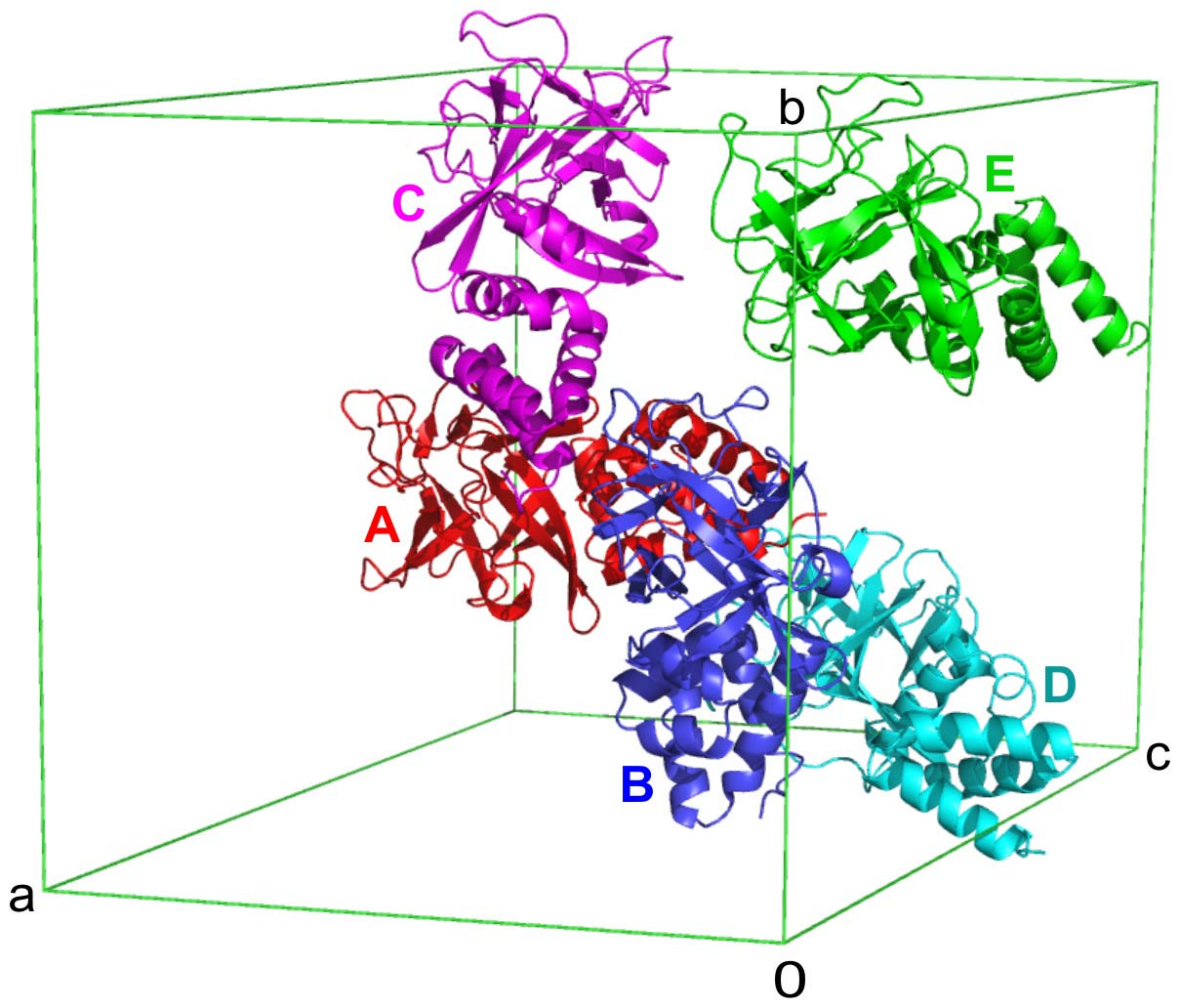
Crystal packing of Bmlp6. The asymmetric unit of Bmlp6 (shown with unit cell outline) is composed of five protein molecules, A–E, represented by different colors.

The asymmetric unit of Bmlp6 is comprised of five protein molecules denoted A, B, C, D and E (Fig. 4). The r.m.s.d. values for all pairwise Cα superpositions of the monomers are very small (ranging from 0.18 to 0.27 Å) indicating an essentially identical folding pattern. The interactions between the monomers in the crystal lattice are weak with no indication of possible quaternary structure, as established by the PISA server [49]. This observation is in agreement with the results of gel filtration and dynamic light scattering (DLS) experiments (not shown), which confirmed the monomeric state of Bmlp6 in solution.

According to the Matthews coefficient and solvent-content analysis [50], the asymmetric unit of the Bmlp6 crystal could contain from 4 to 6 protein molecules. The final solution of the phase problem corresponds to a model comprising five Bmlp6 monomers in the asymmetric unit, with a Matthews coefficient of 2.62 Å^3^/Da and solvent content of 53.1%. Such a composition of the asymmetric unit is quite rare in protein crystallography, and especially in the space group *P*2_1_. Four of the Bmlp6 molecules (A, B, C, D) are arranged in such a manner that the NTD of one monomer (e.g. C) interacts with residues at the NTD/CTD junction of the following (e.g. A) monomer (Fig. 4), forming a chain along the crystallographic **b** axis. The chain is composed of only four protein molecules (C, A, B, D) and is not continuous, meaning that the terminal monomers (D and C) do not interact with their symmetry-related copies. Monomer E is located at the side of the A–D chain and shows even weaker intermolecular interactions than the chain-forming monomers. It is of note that the electron density maps for molecule E were of lower quality, probably because of a higher propensity for disorder allowed by less tight crystal packing. This is also reflected in the average B-factor values calculated in the program BAVERAGE from the CCP4 suite [51] for each monomer separately. The values are 23.4, 21.3, 21.9, 27.5 and 36.0 Å^2^ for monomers A, B, C, D and E, respectively.

At 1.8 Å resolution, we were able to identify in the solvent area of the crystal a number of small molecules from the crystallization buffer. Specifically, molecules of MES, isopropanol and fragments of PEG were mainly located at the protein surface.

### Structural comparison of Bmlp6, Bmlp7 and Bmlp3

Bmlp6 shares with Bmlp7 and Bmlp3 ∼65% similar and ∼49% identical residues (Fig. 3A), which is significantly lower than for the Bmlp3/Bmlp7 pair (97% similarity, 94% identity). When chain A of the Bmlp6 crystal structure was superposed (Fig. 3B) with chain A of Bmlp7 (PDB ID: 4EFP), and with chains A and B of Bmlp3 (PDB ID: 4IY9), the r.m.s.d. values for the Cα atoms were 0.8, 0.8 and 1.0 Å, respectively. In terms of secondary structure elements, the main differences are observed for the loops present in the CTD. As reported previously [35], the most flexible loop (Fig. 3C) is formed by residues 106-116 in Bmlp3 and Bmlp7 (110–120 in Bmlp6). Another significant difference is the conformation of loop 159–171 in Bmlp6 (155–165 in Bmlp3 and Bmlp7), Fig. 3D. It is of note that two cysteine residues separated by only one residue (Cys164, Cys166) present in this loop in Bmlp6, form a disulfide bridge, rendering this loop much more rigid. Bmlp3 and Bmlp7 do not contain any disulfide bonds, and the corresponding loop does not contain any cysteine residues and is slightly shorter in those two proteins. All Bmlp6-CTD loops have the same conformation in each of the five monomers present in the asymmetric unit.

**Figure 5:**
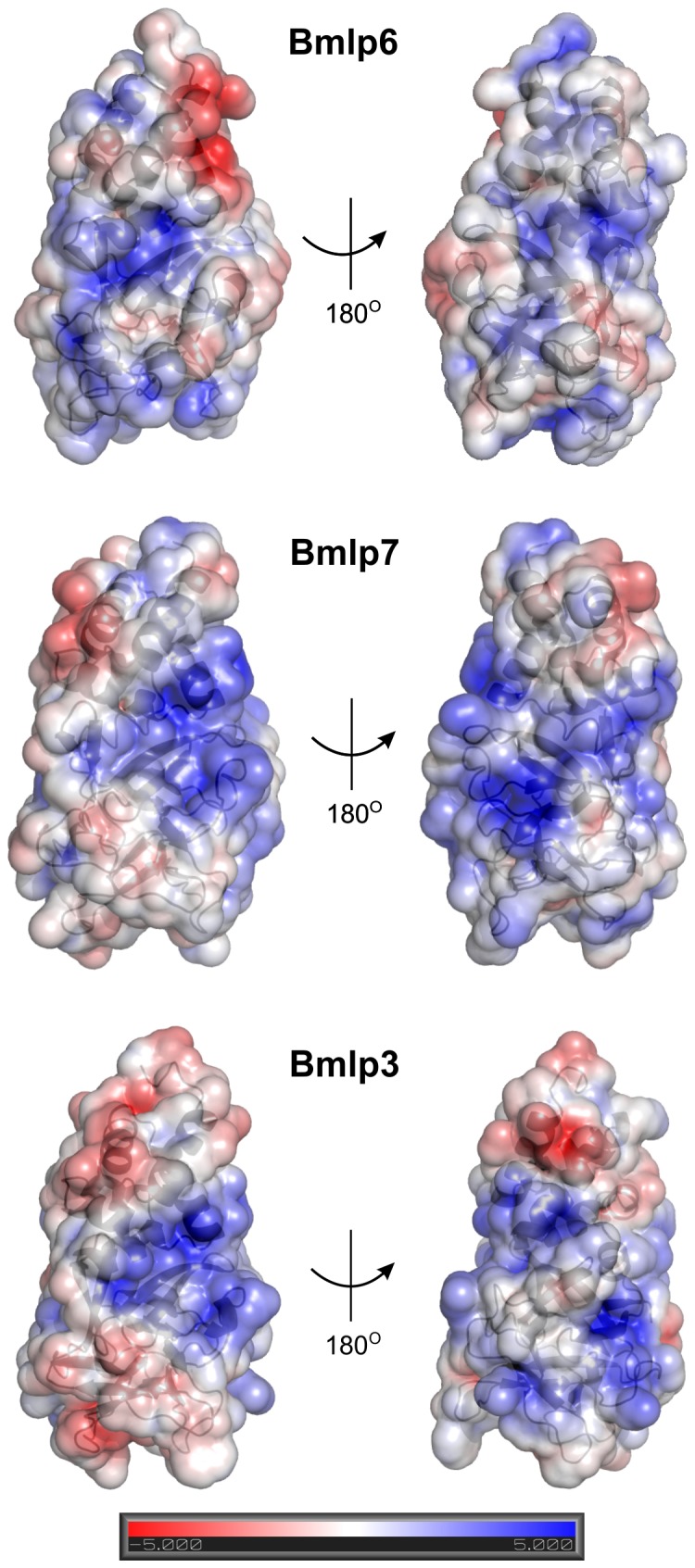
Electrostatic surface potential of Bmlp6, Bmlp7 and Bmlp3 at pH 6.5. Electrostatic surface potential of Bmlp6, Bmlp7 and Bmlp3 was calculated using the *APBS* algorithm [52] and the *PDB2PQR* program [53], [54] at pH 6.5, which is the physiological pH of silkworm hemolymph. The protein surfaces are shown in the same orientation, in two different views for each protein. The positive and negative charges are colored blue and red, respectively, according to the scale. The *APBS* writes out the electrostatic potential in dimensionless units of k_b_Te_c_^−1^ where k_b_ is Boltzmann's constant, T is the temperature of calculation and e_c_ is the charge of an electron [52].

Additionally, the protein surface potential of Bmlp6, Bmlp3 and Bmlp7 was compared (Fig. 5). The Poisson-Boltzmann electrostatic potential on the molecular surface of chains A of the crystallographic models of these proteins was calculated using the *APBS* algorithm [52] and the *PDB2PQR* program [53], [54]. The electrostatic potential calculations were performed at pH 6.5, which is the physiological pH of silkworm hemolymph [55]. Prior to the calculations, the proper side-chain protonation states of the analyzed proteins were determined in PropKa [56]. The patterns of the electrostatic potential on the surfaces of Bmlp3 and Bmlp7 are very similar, whereas significant differences are observed for Bmlp6 (Fig. 5). For instance, the area between helices α1 and α3 is negatively charged in Bmlp6, while not in Bmlp3 and Bmlp7.

**Figure 6:**
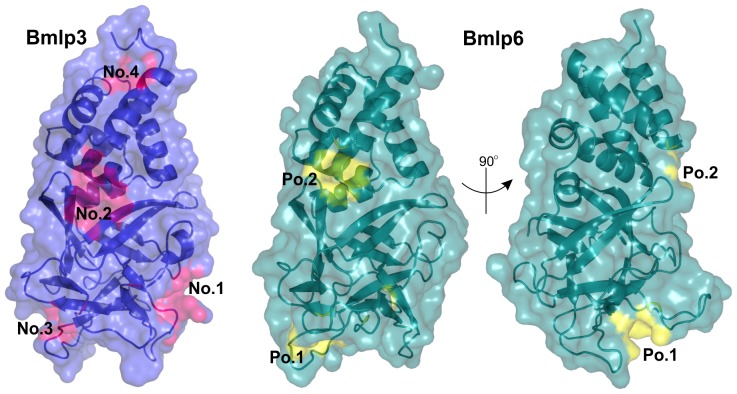
Potential binding sites of Bmlp3 and Bmlp6. Potential binding sites of Bmlp3 (blue/pink) and of Bmlp6 (green/yellow) were marked as No. 1–4 and Po.1–2, respectively. Two different views of Bmlp6 are shown to present both cavities.

Chain A of Bmlp6 was also analyzed for potential binding cavities using the CASTp [57] and metaPocket 2.0 [58] servers. There are only two potential cavities in Bmlp6, denoted Po.1 and Po.2 (Fig. 6), in contrast to Bmlp3 and Bmlp7, where three cavities (No. 1–3) are present. Since the analysis produced identical results for the latter two proteins, Bmlp3 [35] will be used for comparison. The cavity Po.2 of Bmlp6 is found at a similar location to pocket No. 2 of Bmlp3, between the NTD and CTD. Moreover, it is formed by corresponding residues in both proteins. However, the cavity No. 2 of Bmlp3 is larger and involves more CTD residues. The pocket Po.1 of Bmlp6 is formed by different residues than cavity No. 3 of Bmlp3, although both pockets are located in a similar region (in the CTD) of the structure. Moreover, the two potential pockets of Bmlp6 (Table 2) are small, with a volume of ∼90 Å^3^, while the largest cavity of Bmlp3 has a volume of ∼400 Å^3^. The pocket Po.1 has a small surface area of 86 Å^2^, although it has a slightly larger volume. It is formed mainly by hydrophilic residues. Interestingly, these residues belong to the two loops discussed above, the flexible loop 110–120 and the Bmlp6-characteristic loop 159–171. It is plausible that this cavity might undergo some conformational changes upon ligand binding. The cavity Po.2 covers an area of 101 Å^2^ and is also hydrophilic. Both Bmlp6 cavities are located in positively charged areas.

**Table 2:** Potential binding sites of the Bmlp6 protein.

Cavity	Area [Å^2^]	Volume [Å^3^]	Location in the structure	Residues forming the cavity
Po.1	86	97	CTD	N112, S114, E116, S160, T161, T162, D170, R171, Y174
Po.2	101	84	NTD-CTD	G25, Y27, R59, N60, E63, N142, R143

### Structural differences of 30-kDa LPs as potential basis of functional diversity

As mentioned above, the silkworm genome contains 46 genes of 30-kDa LPs, 24 of which are classified as typical 30-kDa LPs [11]. 30-kDa LPs have been demonstrated to bind physiological ligands belonging to at least two groups, namely lipids and carbohydrates [16], [18], [19]. The 30-kDa proteins from silkworm hemolymph have been classified as lipoproteins according to specific lipid staining [16]. On the other hand, the 30-kDa LPs are involved in the immune response pathway: they recognize and bind β-glucans present on the surface of fungal cells [18], [19]. Moreover, the insect hemolymph contains a number of chemical compounds [59], and some of them might be binding partners of the 30-kDa LPs as well. Bmlp3, Bmlp6 and Bmlp7 share a similar fold and it is very likely that all 24 typical LPs have the same fold. However, the high number of “isoforms” of these proteins in one organism is suggestive of different ligand specificity of each particular 30-kDa LP member. Moreover, it might be speculated that the proteins play in the insect immune response a role similar to human antibodies. For example, each of the 30-kDa LPs could be specific to a different carbohydrate pattern.

It is of note that even with the crystal structures of only three members of the 30-kDa LP family determined and compared, many differences are already obvious, such as: (i) different conformation of CTD loops, (ii) different electrostatic surface potential, and (iii) different potential binding cavities. It is also possible that the 30-kDa LPs can interact with other silkworm proteins and that the structural differences enable them to recognize different binding partners.

Taken together, the high number of 30-kDa LP genes, their diverse expression patterns and structural differences, all seem to indicate that members of the 30-kDa LP family might represent a wide range of specificities for different physiological ligands present in the hemolymph, as well as different specificity for protein partners, implicating different roles in silkworm physiology.

## Conclusions

Bmlp6 is the third most abundant 30-kDa LP in the hemolymph of the silkworm fifth instar larvae. Its identification could be accomplished mainly by sequencing according to electron density maps. The crystal structure of 1.8 Å resolution shows five copies of a two-domain molecule with a typical 30-kDa LP fold. The structure demonstrates that Bmlp6 differs from Bmlp3 and Bmlp7 in the conformation of loops, the electrostatic surface potential, and potential binding cavities. Taking into account the large number of 30-kDa LP genes and the structural differences between the proteins, the question about the exact physiological role of each particular 30-kDa LP member remains open. Our studies suggest functional diversity, fine-tuned to the structural differences, which exist despite the overall similarity.
